# Curvature-Influenced Electrocatalytic NRR Reactivity by Heme-like FeN_4_-Site on Carbon Materials

**DOI:** 10.3390/molecules30081670

**Published:** 2025-04-08

**Authors:** Yajie Meng, Ziyue Huang, Xi Chen, Yingqi Li, Xueyuan Yan, Jiawei Xu, Haiyan Wei

**Affiliations:** 1 Fujian Institute of Research on the Structure of Matter, Chinese Academy of Sciences, Fuzhou 350002, China; mengyajie@fjirsm.ac.cn; 2 School of Chemical Sciences, University of Chinese Academy of Sciences, Beijing 100049, China; 3 Jiangsu Key Laboratory of Biofunctional Materials, School of Chemistry and Materials Science, Ministry-of-Education Key Laboratory of Numerical Simulation of Large-Scale Complex Systems, Nanjing Normal University, Nanjing 210023, China; 231112019@njnu.edu.cn (Z.H.); xichenchem@njfu.edu.cn (X.C.); yingqilichem@gmail.com (Y.L.); 4 College of Chemistry & Chemical Engineering, Weifang University, Weifang 261061, China; 5 Physical and Theoretical Chemistry Laboratory, Department of Chemistry, University of Oxford, Oxford OX1 3QZ, UK

**Keywords:** electrocatalytic NRR, carbon nanotube, single-atom catalyst, ammonia synthesis, surface distortion

## Abstract

Two-dimensional carbon materials and their derivatives are widely applied as promising electrocatalysts and supports of single-atom sites. Theoretical investigations of 2D carbon materials are usually based on planar models, yet ignore local curvature brought on by possible surface distortion, which can be significant to the exact catalytic performance as has been realized in latest research. In this work, the curvature-influenced electrocatalytic nitrogen reduction reaction (NRR) reactivity of heme-like FeN_4_ single-atom site was predicted by a first-principle study, with FeN_4_-CNT(*m*,*m*) (*m* = 5~10) models adopted as local curvature models. The results showed that a larger local curvature is favored for NRR, with a lower limiting potential and higher N_2_ adsorption affinity, while a smaller local curvature shows lower NH_3_ desorption energy and is beneficial for catalyst recovery. Using electronic structures and logarithm fitting, we also found that FeN_4_-CNT(5,5) shows an intermediate-spin state, which is different from the high-spin state exhibited by other FeN_4_-CNT(*m*,*m*) (*m* = 6~10) models with a smaller local curvature.

## 1. Introduction

Two-dimensional carbon materials, including but not limited to graphene [1], graphdiyne [2,3], and their derivatives [4], are well developed as promising electrocatalysts and supports of single-atom sites [5,6,7]. Considering their flexibility, synthesized 2D carbon materials do not maintain ideally planar structures and form locally distorted surfaces, affecting their catalytic performance in electrochemistry [8,9]. In most cases, planar structures are used in theoretical investigations for the sake of simplification [10,11,12,13], while possible influence brought about by a local curvature originated from distortion is rarely discussed compared with planar models. Recent works have gradually started to investigate curvature-influenced electrocatalytic processes like the oxygen reduction reaction (ORR) [14,15], hydrogen evolution reaction (HER) [16], CO_2_ reduction reaction (CO_2_RR) [17,18], and peroxymonosulfate (PMS) activation [19]. Previous research results have shown that the curvature of surface structures makes a notable difference to catalytic performance, and by adjusting the local curvature, a higher catalytic efficiency and new reaction behavior can be achieved, as have been well summarized in review articles [20,21,22,23].

**Figure 1:**
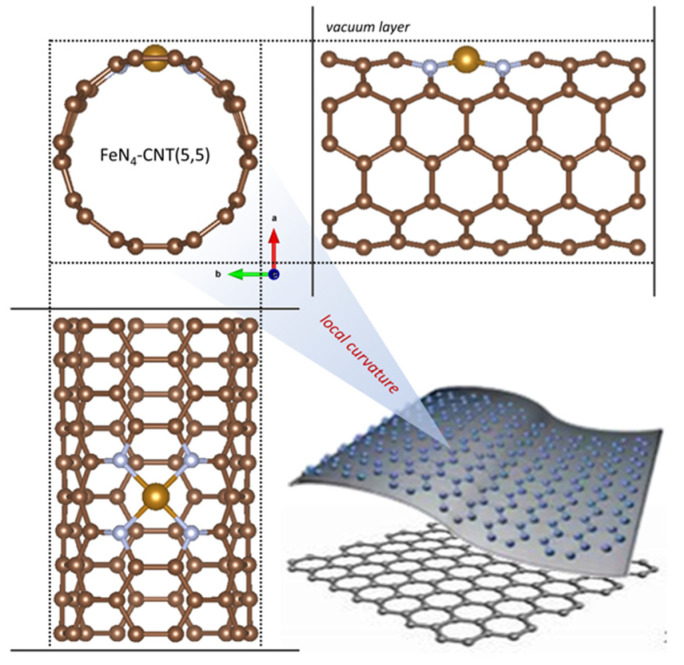
Top and side view of FeN_4_-CNT(5,5) as an example. Fe, C, and N are displayed in gold, brown, and blue, respectively.

The electrocatalytic nitrogen reduction reaction (NRR) is an environmentally friendly and sustainable method for synthesizing ammonia (NH_3_) and has gained increasing attention in recent years, and more and more efficient electrocatalysts that activate N_2_ molecules have been discovered and designed for NH_3_ production [24,25,26]. Experimentally, Ziming Zhao et al. synthesized a high-efficiency NRR electrocatalyst, 2D C_3_N_4_-NV, and its abundant N vacancy facilitates N_2_ adsorption and activation [27]. Compared with Bi nanoparticles, Bi quantum dots (Bi QDs) show a high NH_3_ yield, and the size of Bi particles obviously affects their NRR catalytic performance [28]. In addition, plenty of research been conducted on the theoretical investigation of the electrocatalytic NRR based on planar models [29,30,31,32], yet how and to what extent curvature influences NRR performance is rarely discussed. In this work, by constructing heme-like FeN_4_ single-atom sites on carbon nanotubes (CNTs) with different chiral indices (*m*,*m*), where *m* = 5~10 (Figure 1), the curvature-influenced electrocatalytic NRR reactivity was investigated via first-principle calculations. The larger chiral index *m* corresponds to the gradual increase in the nanotube diameter and decrease in the local curvature. Our results showed that a larger curvature is favored for N_2_ adsorption and exhibits lower limiting potential, while a smaller curvature is beneficial for NH_3_ desorption so as to help catalyst recovery. Moreover, as a model for large curvature, FeN_4_-CNT(5,5) shows a different electronic structure compared with other models.

## 2. Results and Discussion

**Scheme 1:**
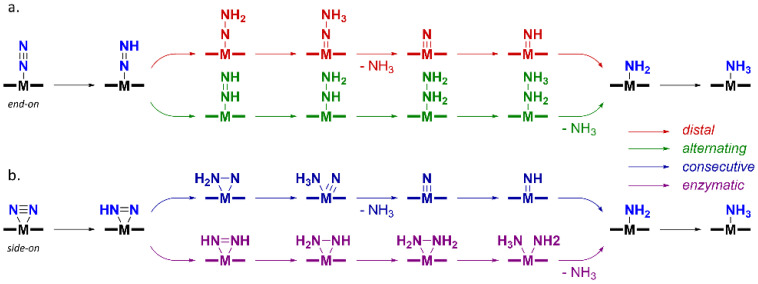
Common N_2_ adsorption configurations and reaction pathways of electrocatalytic NRR on heme-like single-atom site (M: metal). (**a**) Distal and alternating pathways; (**b**) consecutive and enzymatic pathways.

The electrocatalytic NRR involves six hydrogenation steps, which is generally considered to have distal and alternating pathways on single-atom sites with end-on N_2_ adsorption configuration, as depicted in Scheme 1a. Since we are applying an FeN_4_-site as metallic center, which has almost planar heme-like coordination form and side-on N_2_ adsorption configuration is generally considered to be unfavored, we therefore excluded consecutive and enzymatic pathways (Scheme 1b) started with the side-on configuration in the following discussion.

**Figure 2:**
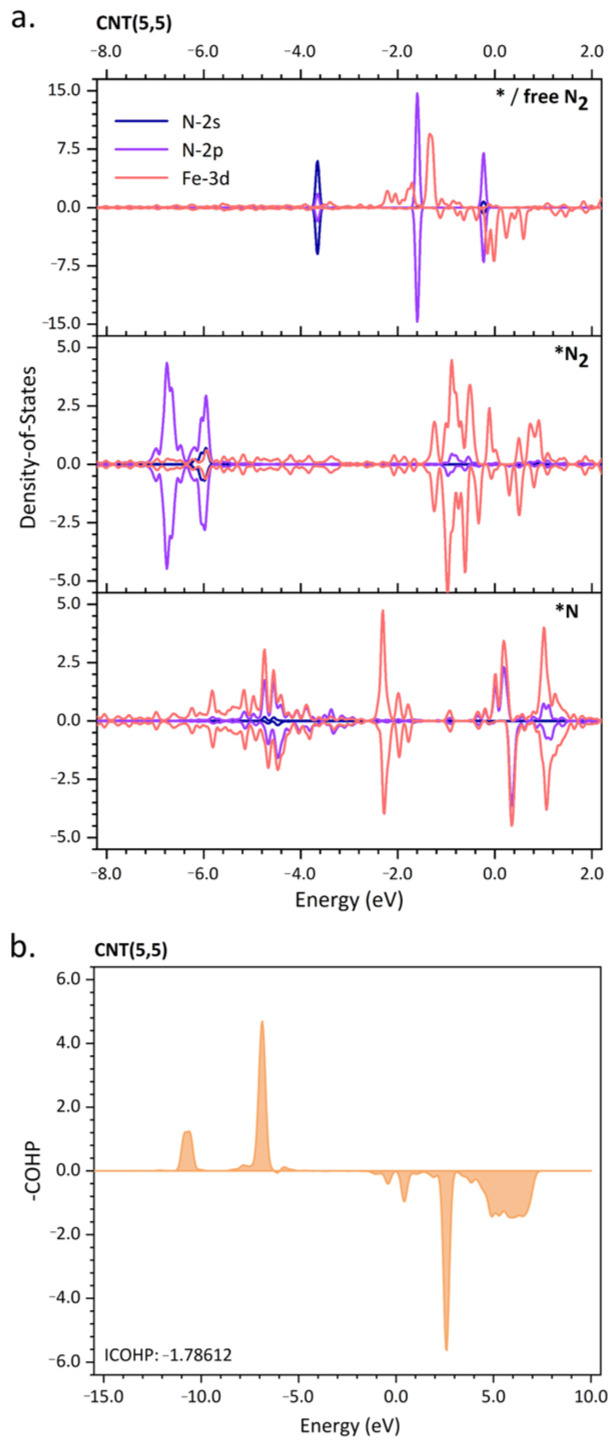
(**a**) Projected density-of-states (PDOS) results of free N_2_, FeN_4_-CNT(5,5), N_2_@, and N@FeN_4_-CNT(5,5) as examples; (**b**) COHP results of N_2_@FeN_4_-CNT(5,5) as an example.

We first look into the influence on the electronic structure of a metallic center brought about by a local curvature. Many studies have confirmed that the Fe center is the active site of FeN_4_-doped carbon materials [33,34]. As shown in Figure 2a and Figure S1, the projected density of states (PDOS) of Fe 3d orbitals in FeN_4_-CNTs, as well as 2s and 2p orbitals of free and absorbed N_2_, were calculated to reveal the electronic structure of FeN_4_-CNTs and related catalytic intermediates. The PDOS of Fe 3d orbitals in FeN_4_-CNTs is mainly distributed near the Fermi level with a notable spin asymmetry of different spin states, indicating a high-spin metallic center, regardless the local curvature of carbon materials.

As is displayed in Figure 2a and Figure S1, the adsorbed N_2_ retained its spin-symmetry in *N_2_ intermediate, while the PDOS of the Fe 3d orbitals becomes more spin-symmetric and less magnetic, giving evidence to a low-spin metallic center, where N_2_ shows a feature of π-acid strong-field ligand. Therefore, FeN_4_-CNTs are considered to have strong affinity to N_2_ adsorption, which is essential to N_2_ activation and following N_2_ reduction steps. A similar low spin state is observed for all *N intermediates, with stable Fe-N covalent bond formed to stabilize the highly active single nitrogen atom, enabling N-N bond cleavage in the distal pathway. Comparing the DOS of free and adsorbed N_2_, it can be found that the valence orbitals of N_2_ exhibit a significant decrease and move down after adsorption. In free N_2_, the N-N σ* orbital is located near the Fermi level, while this signal shows a significant decrease in *N_2_, suggesting that the N-N σ bond is weakened after N_2_ adsorption, promoting the activation of N-N bond. In addition, the overlap of Fe 3d orbitals and 2p orbitals of N_2_ at −6.0 to −8.0 eV indicates the d-π orbital interaction. Similar results on N_2_ adsorption electronic structure are observed by the crystal orbital Hamiltonian population (COHP) calculation. As shown in Figure 2b and Figure S2, overlaps in the range of −12.0~−5.0 eV show two positive -COHP regions, corresponding to the stable bonding structure revealed by a change in the PDOS. The integral crystal orbital Hamilton population (ICOHP) value reflects the Fe-N bond strength between FeN_4_-CNTs and N_2_, where the larger -ICOHP value represents a stronger interaction between the catalyst and N_2_ [35,36]. In FeN_4_-CNT(5,5) to (10,10), the -ICOHP value gradually increases, which means that the binding of N_2_ to the catalyst gradually increases.

**Figure 3:**
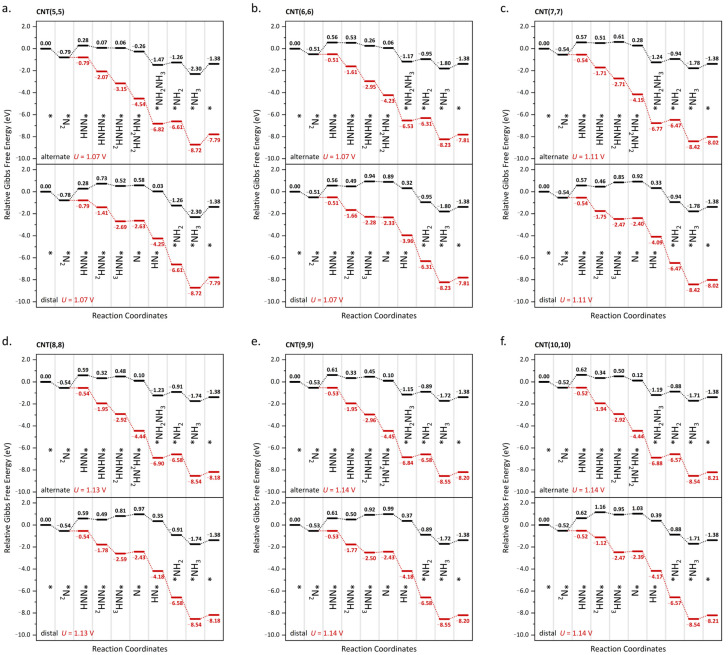
Gibbs free energy diagrams of alternating and distal NRR pathways on FeN_4_-CNTs with different sizes.

**Figure 4:**
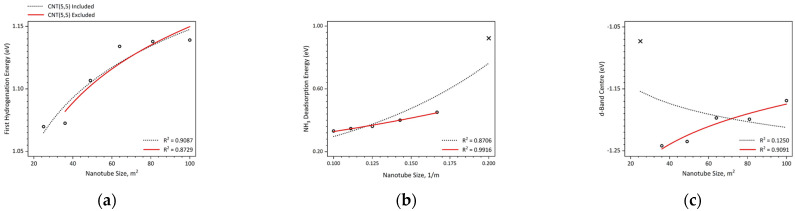
Logarithm relationships between (**a**) the first hydrogenation energy (Δ*G*_1_, eV) and local curvature revealed by nanotube size (*m*^2^); (**b**) NH_3_ desorption energy and 1/*m*; (**c**) d-band center of Fe and *m*^2^.

Based on the designed adsorption models, both the distal and alternating pathways were calculated for FeN_4_-CNTs to investigate how the NRR performance can be affected by the local curvature of carbon materials, the Gibbs free energy profiles of which are displayed in Figure 3. It can be seen that regardless of the local curvature, both the distal and alternating pathways share the same rate-determining step, which is the first hydrogenation step (*N_2_ → *NNH), requiring an increasing limiting potential of 1.07 to 1.14 eV from FeN_4_-CNT(5,5) to (10,10). This points out that the electrocatalytic capacity of the FeN_4_-site on distorted carbon materials gradually decreases with a lower local curvature. Considering that the smaller local curvature gives a more similar electronic structure and catalytic properties compared with ideally planar graphene-based single-atom site, as shown in Figure 4a, a logarithm relationship was found between the first hydrogenation energy and the local curvature of carbon materials (evaluated by nanotube size, *m*). Including or excluding FeN_4_-CNT(5,5) in the logarithm fitting does not brought much difference to the *R*^2^ value, showing that the FeN_4_-CNT(5,5) model with a large local curvature is still within a reasonable range of catalytic performance, and therefore, it is acceptable for modulating the influence on the electrocatalytic NRR brought on by the local curvature.

The desorption energy of NH_3_ is crucial to the recovery of catalyst, as shown in Figure 3, and as the local curvature gets smaller from FeN_4_-CNT(5,5) to (10,10) models, the desorption energy of NH_3_ decreases from 0.92 to 0.33 eV, showing that a smaller local curvature is favored by NH_3_ desorption and the catalyst recovery process. We can still find a logarithmic relationship between the NH_3_ desorption energy and local curvature, as displayed in Figure 4b; including the FeN_4_-CNT(5,5) model in the fitting candidates gives an *R*^2^ value of 0.8706, while excluding the FeN_4_-CNT(5,5) model gives an *R*^2^ value of 0.9916. This indicates that a different adsorption electronic structure is formed in the NH_3_@FeN_4_-CNT(5,5) intermediate, compared with other FeN_4_-CNT models. By analyzing the PDOS results (Figure 2a and Figure S1), we may find that the PDOS diagrams of *N_2_ and *N intermediates are almost identical to each other, while the PDOS of Fe 3d orbitals in FeN_4_-CNT(5,5) has a slight difference compared with other FeN_4_-CNT models. The spin-up contribution of FeN_4_-CNT(5,5) is smaller than that in FeN_4_-CNT(*m*,*m*) (*m* = 6~10) models; therefore, compared with the high-spin metallic center in *m* = 6~10 models, FeN_4_-CNT(5,5) shows an intermediate-spin state. Considering that the Fe atom of the FeN_4_-site on carbon materials should have a formal oxidation state of +3 [37], the high-spin state has its all 3d orbitals singularly occupied, therefore forming an adsorption structure determined by the interaction when coordinated by the weak ligand NH_3_. Meanwhile, the intermediate-spin state of FeN_4_-CNT(5,5) has an empty 3d orbital, and can form effective adsorption towards NH_3_, resulting in significantly higher NH_3_ desorption energy in FeN_4_-CNT(5,5).

Correspondingly, the d-band center calculation of FeN_4_-CNT models reveals a similar conclusion. As shown in Figure 4c, the logarithm fitting of the d-band center with local curvature (evaluated by *m*^2^) shows that excluding FeN_4_-CNT(5,5) from fitting candidates gives a well-fitted relationship with an *R*^2^ value of 0.9091. Meanwhile, including the FeN_4_-CNT(5,5) results in the *R*^2^ value of 0.1250 therefore shows that the electronic structure of FeN_4_-CNT(5,5) is highly different from other FeN_4_-CNT models. This is in consistent with the PDOS results.

Adjusting the curvature of carbon materials can change the adsorption energy and energy barriers of the electrocatalytic process, which is an effective method to optimize the electrocatalytic performance of the catalysts [22]. Our work investigates the regulation of the curvature-influenced NRR catalytic reactivity of carbon materials at the atomic scale and analyzes the internal reasons for the curvature influence: a larger local curvature is favored for NRR, and a smaller local curvature shows lower NH_3_ desorption energy, an electronic structure analysis shows that the metal centers of FeN_4_-CNT(*m*,*m*) (*m* = 5~10) models are in intermediate- and high-spin states. These results are complementary to the electrocatalytic performance of FeN_4_-CNTs and provide reliable theoretical guidance for the optimization and modification of the electrocatalytic performance of carbon materials.

Moreover, we should also point out that, in some studies, a linear relationship was used to reveal the relationship between the curvature and catalytic performance [38], while we suggest that any catalytic performance cannot keep increasing/decreasing with the curvature. This is because the limitation has already been established by the graphene-based single-atom catalytic site (with no curvature). Therefore, any catalytic performance will finally converge to that in a graphene-based site, pointing out that a function type with a converging behavior should be applied instead of a linear relationship. That is the reason we used logarithm fitting in Figure 4.

## 3. Computational Methods

All the calculations in this work were performed using the spin-polarized density functional theory (DFT) method of the Vienna Ab initio Simulation Package (VASP) [39]. The Perdew–Burke–Ernzerh (PBE) functional of generalized gradient approximation (GGA) was utilized to describe the electron exchange–correlation interaction [40]. The projector-augmented wave (PAW) method was applied to describe the ion–electron interaction and a plane-wave cutoff energy of 450 eV was employed in all calculations. Grimme’s DFT-D3 dispersion correction was adopted to consider van der Waals interactions [41]. The Brillouin zone integration was performed with a 1 × 1 × 5 grid centered at the gamma (*Γ*) point [42]. The convergence threshold for energy and force was 10^−5^ eV and 0.02 eV·Å^−1^, respectively. The space in *a*- and *b*-axes was set to 30.0 Å to prevent the interaction introduced by the periodic boundary condition. The crystal orbital Hamilton population (COHP) was used to measure the bonding between atoms by using the concept of the LOBSTER code [43].

The Gibbs free energy of the intermediates was calculated using the computational hydrogen electrode (CHE) model proposed by Nørskov et al. [44]. Based on the model, the reaction Gibbs free energy (Δ*G*) of each elementary step can be defined by Equation (1):

(1) $$\begin{array}{c} \Delta G=\Delta E-T\Delta S+\Delta \mathrm{ZPE}+\Delta G_{U}+\Delta G_{\mathrm{pH}} \end{array}$$

where Δ*E* is the electronic energy difference for reactions directly obtained from DFT calculations. ΔZPE and Δ*S* are the differences between the adsorbed species and the gas phase molecules in zero-point energy and entropy, respectively. Δ*G_U_* = −*eU* is the free energy contribution related to applied potential *U*, which is determined by *U* = −Δ*G*_PDS_/*e*, where Δ*G*_PDS_ is the free energy change in potential-determining step (PDSs). Δ*G*_pH_ refers to the correction of free energy caused by pH (Δ*G*_pH_ = 2.303 *k*_B_·pH), and the pH value is set to zero in this work [45].

The d band center (*ε*_d_) is a well-defined electronic descriptor for metal-contained catalysts and is given by Equation (2) [46,47]:

(2) $$\begin{array}{c} \varepsilon_{d}=\frac{\int n_{d}\left(\varepsilon \right)\varepsilon \;d\varepsilon }{\int n_{d}\left(\varepsilon \right)\;d\varepsilon } \end{array}$$

where *ε*, d*ε*, *n*_d_(*ε*) represent the energy level, differential of energy, and density of states (DOS) corresponding to energy levels, respectively.

## 4. Conclusions

The catalytic performance influenced by the local curvature of 2D carbon materials and their derivatives has been noticed in recent research, urging theoretical research to focus on curvature models beyond ideal planar models. In this work, the electrocatalytic NRR reactivity curvature-influenced by the heme-like FeN_4_ single-atom site was investigated by a first-principle study, with FeN_4_-CNT(*m*,*m*) (*m* = 5~10) models as theoretical models for possible surface distortion. Our results show that a larger local curvature gives a lower limiting potential and higher N_2_ adsorption affinity, which is favored by the high-efficiency electrocatalytic NRR. Meanwhile, a smaller local curvature also has a minor advantage over NH_3_ desorption, which contributes to catalyst recovery. These curvature performance trends were well fitted in the logarithm relationships. Moreover, an electronic structure analysis also found that the higher curvature in FeN_4_-CNT(5,5) leads to an intermediate-spin state, which is different from other curvature models with a high-spin state, and influences the binding mode taken by key catalytic intermediates. Our findings pointed out that curvature influence should be well considered in future theoretical and also experimental studies to discover new electrocatalysts or to make modifications based on known ones.

## Data Availability

The data presented in this manuscript are available in the Supplementary Materials.

## Supplementary Materials

The following supporting information can be downloaded at: https://www.mdpi.com/article/10.3390/molecules30081670/s1, Figures S1 and S2: PDOS and COHP results; Table S1: Optimized cell parameters of FeN_4_-CNT; Tables S2–S13: Thermochemical data of catalytic intermediates of FeN_4_-CNT(*m*,*m*) (*m* = 5~10) models.

## References

[1] Fang Q., Shen Y., Chen B. (2015). Synthesis, decoration and properties of three-dimensional graphene-based macrostructures: A review. Chem. Eng. J..

[2] Gao Y., Cai Z., Wu X., Lv Z., Wu P., Cai C. (2018). Graphdiyne-supported single-atom-sized Fe catalysts for the oxygen reduction reaction: DFT predictions and experimental validations. ACS Catal..

[3] Gao X., Liu H., Wang D., Zhang J. (2019). Graphdiyne: Synthesis, properties, and applications. Chem. Soc. Rev..

[4] Cheng Y., Zhao S., Li H., He S., Veder J.-P., Johannessen B., Xiao J., Lu S., Pan J., Chisholm M.F. (2019). Unsaturated edge-anchored Ni single atoms on porous microwave exfoliated graphene oxide for electrochemical CO_2_. Appl. Catal. B Environ..

[5] Mitchell S., Pérez-Ramírez J. (2020). Single atom catalysis: A decade of stunning progress and the promise for a bright future. Nat. Commun..

[6] Wang A., Li J., Zhang T. (2018). Heterogeneous single-atom catalysis. Nat. Rev. Chem..

[7] Cheng N., Zhang L., Doyle-Davis K., Sun X. (2019). Single-atom catalysts: From design to application. Electrochem. Energy Rev..

[8] Katiyar A.K., Hoang A.T., Xu D., Hong J., Kim B.J., Ji S., Ahn J.-H. (2024). 2D Materials in Flexible Electronics: Recent Advances and Future Prospectives. Chem. Rev..

[9] Yu W., Gong K., Li Y., Ding B., Li L., Xu Y., Wang R., Li L., Zhang G., Lin S. (2022). Flexible 2D Materials beyond Graphene: Synthesis, Properties, and Applications. Small.

[10] Fei H., Dong J., Feng Y., Allen C.S., Wan C., Volosskiy B., Li M., Zhao Z., Wang Y., Sun H. (2018). General synthesis and definitive structural identification of MN_4_C_4_ single-atom catalysts with tunable electrocatalytic activities. Nat. Catal..

[11] Zhong W., Yue J., Zhang R., Huang H., Huang H., Shen Z., Jiang L., Xu M., Xia Q., Cao Y. (2024). Screening of Transition Metal Supported on Black Phosphorus as Electrocatalysts for CO_2_ Reduction. Inorg. Chem..

[12] Xia J., Xu J., Yu B., Liang X., Qiu Z., Li H., Feng H., Li Y., Cai Y., Wei H. (2024). A Metal–Sulfur–Carbon Catalyst Mimicking the Two-Component Architecture of Nitrogenase. Angew. Chem. Int. Ed..

[13] You X., Xu J., Zhuang Z., Xia J., Wang S., Wei H., Li Y., Cai Y., Xiang H., Yu B. (2024). Ru-Ni alloy nanosheets as tandem catalysts for electrochemical reduction of nitrate to ammonia. Nano Res..

[14] Liang Z., Kong N., Yang C., Zhang W., Zheng H., Lin H., Cao R. (2021). Highly curved nanostructure-coated Co, N-doped carbon materials for oxygen electrocatalysis. Angew. Chem..

[15] Wang D.-W., Su D. (2014). Heterogeneous nanocarbon materials for oxygen reduction reaction. Energy Environ. Sci..

[16] Wang Z., Gao S., Liu X., Chen X., Zhang X., Sa R., Li Q., Sun C., Ma Z. (2023). Defective SiC nanotube based single-atom catalysts for electrocatalytic nitrogen fixation with curvature effect. Mol. Catal..

[17] De Luna P., Quintero-Bermudez R., Dinh C.-T., Ross M.B., Bushuyev O.S., Todorović P., Regier T., Kelley S.O., Yang P., Sargent E.H. (2018). Catalyst electro-redeposition controls morphology and oxidation state for selective carbon dioxide reduction. Nat. Catal..

[18] Yan X., Duan C., Yu S., Dai B., Sun C., Chu H. (2024). Recent advances on CO_2_ reduction reactions using single-atom catalysts. Renew. Sustain. Energy Rev..

[19] Shi H., He Y., Li Y., He T., Luo P. (2022). Efficient degradation of tetracycline in real water systems by metal-free g-C_3_N_4_ microsphere through visible-light catalysis and PMS activation synergy. Sep. Purif. Technol..

[20] Wang Y., Bao Z., Shi M., Liang Z., Cao R., Zheng H. (2022). The role of surface curvature in electrocatalysts. Chem. A Eur. J..

[21] Mohamed A.G.A., Huang Y., Xie J., Borse R.A., Parameswaram G., Wang Y. (2020). Metal-free sites with multidimensional structure modifications for selective electrochemical CO_2_ reduction. Nano Today.

[22] Wen H., Zhao Z., Luo Z., Wang C. (2024). Unraveling the Impact of Curvature on Electrocatalytic Performance of Carbon Materials: A State-of-the-Art Review. ChemSusChem.

[23] Liu Y., Wu Z., Gu C., Chen J., Zhu Y., Wang L. (2024). Curved Structure Regulated Single Metal Sites for Advanced Electrocatalytic Reactions. Small.

[24] Li M., Huang H., Low J., Gao C., Long R., Xiong Y. (2019). Recent progress on electrocatalyst and photocatalyst design for nitrogen reduction. Small Methods.

[25] Guo C., Ran J., Vasileff A., Qiao S.-Z. (2018). Rational design of electrocatalysts and photo (electro) catalysts for nitrogen reduction to ammonia (NH_3_) under ambient conditions. Energy Environ. Sci..

[26] Bao D., Zhang Q., Meng F.-L., Zhong H.-X., Shi M.-M., Zhang Y., Yan J.-M., Jiang Q., Zhang X.-B. (2017). Electrochemical Reduction of N_2_ under Ambient Conditions for Artificial N_2_ Fixation and Renewable Energy Storage Using N_2_/NH_3_ Cycle. Adv. Mater..

[27] Zhao Z., Long Y., Luo S., Luo Y., Chen M., Ma J. (2021). Metal-Free C_3_N_4_ with plentiful nitrogen vacancy and increased specific surface area for electrocatalytic nitrogen reduction. J. Energy Chem..

[28] Guo P., Yin F., Ni Z., Shi L., Kofie G. (2025). Efficient Bi catalysts for electrochemical synthesis of ammonia in neutral electrolyte: The effect of Bi particle size on the NRR reaction mechanism. Chem. Eng. J..

[29] Liu P., Fu C., Li Y., Wei H. (2020). Theoretical screening of single atoms anchored on defective graphene for electrocatalytic N_2_ reduction reactions: A DFT study. Phys. Chem. Chem. Phys..

[30] Fu C., Li Y., Wei H. (2021). Double boron atom-doped graphdiynes as efficient metal-free electrocatalysts for nitrogen reduction into ammonia: A first-principles study. Phys. Chem. Chem. Phys..

[31] Yu X., Han P., Wei Z., Huang L., Gu Z., Peng S., Ma J., Zheng G. (2018). Boron-doped graphene for electrocatalytic N_2_ reduction. Joule.

[32] He C., Wu Z.-Y., Zhao L., Ming M., Zhang Y., Yi Y., Hu J.-S. (2019). Identification of FeN_4_ as an efficient active site for electrochemical N2 reduction. ACS Catal..

[33] Chu K., Gu W., Li Q., Liu Y., Tian Y., Liu W. (2021). Amorphization activated FeB_2_ porous nanosheets enable efficient electrocatalytic N_2_ fixation. J. Energy Chem..

[34] Zhang L., Liang P., Man X.-l., Wang D., Huang J., Shu H.-b., Liu Z.-g., Wang L. (2019). Fe, N co-doped graphene as a multi-functional anchor material for lithium-sulfur battery. J. Phys. Chem. Solids.

[35] Dronskowski R., Blöchl P.E. (1993). Crystal orbital Hamilton populations (COHP): Energy-resolved visualization of chemical bonding in solids based on density-functional calculations. J. Phys. Chem..

[36] Deringer V.L., Tchougréeff A.L., Dronskowski R. (2011). Crystal orbital Hamilton population (COHP) analysis as projected from plane-wave basis sets. J. Phys. Chem. A.

[37] Zhang J., Wang Q., Qiu C., Gan L., Ding J., Li F., Wang T., Liu Y., Wang Y., Tao H. (2023). Boosting activity of Fe-N_4_ sites in single-Fe-atom catalysts via S in the second coordination sphere for direct methanol fuel cells. Cell Rep. Phys. Sci..

[38] Sui H., Guo Q., Xiang M., Kong X., Zhang J., Ding S., Su Y. (2024). Theoretical Insights of Curvature Effects of FeN_4_-Doped Carbon Nanotubes on ORR Activity. J. Phys. Chem. Lett..

[39] Kresse G., Hafner J. (1993). Ab initio molecular dynamics for open-shell transition metals. Phys. Rev. B.

[40] Grimme S. (2006). Semiempirical GGA-type density functional constructed with a long-range dispersion correction. J. Comput. Chem..

[41] Blochl P.E. (1994). Projector augmented-wave method. Phys. Rev. B.

[42] Froyen S. (1989). Brillouin-zone integration by Fourier quadrature: Special points for superlattice and supercell calculations. Phys. Rev. B.

[43] Maintz S., Deringer V.L., Tchougréeff A.L., Dronskowski R. (2016). LOBSTER: A tool to extract chemical bonding from plane-wave based DFT. J. Comput. Chem..

[44] Nørskov J.K., Rossmeisl J., Logadottir A., Lindqvist L., Kitchin J.R., Bligaard T., Jonsson H. (2004). Origin of the overpotential for oxygen reduction at a fuel-cell cathode. J. Phys. Chem. B.

[45] Montoya J.H., Tsai C., Vojvodic A., Nørskov J.K. (2015). The challenge of electrochemical ammonia synthesis: A new perspective on the role of nitrogen scaling relations. ChemSusChem.

[46] Takigawa I., Shimizu K.-i., Tsuda K., Takakusagi S. (2016). Machine-learning prediction of the d-band center for metals and bimetals. RSC Adv..

[47] Guo H., Li L., Wang X., Yao G., Yu H., Tian Z., Li B., Chen L. (2019). Theoretical investigation on the single transition-metal atom-decorated defective MoS_2_ for electrocatalytic ammonia synthesis. ACS Appl. Mater. Interfaces.

