# Supplementary material for: Curvature-Influenced Electrocatalytic NRR Reactivity by Heme-like FeN4-Site on Carbon Materials
Source: Molecules. 2025 Apr 8;30(8):1670. doi: 10.3390/molecules30081670 (PMC12029958; doi:10.3390/molecules30081670)
Supplement: Supplementary file 1 [file molecules-30-01670-s001.zip › molecules-3511510-supplementary.pdf]

Supporting Information

Curvature-Influenced Electrocatalytic NRR Reactivity by Heme-Like FeN_4-Site on Carbon Materials

Yajie Meng[1,2], Ziyue Huang[3], Xi Chen[3], Yingqi Li[3], Xueyuan Yan,[4]* Jiawei Xu[3,5]* and Haiyan Wei[3]*

1. Fujian Institute of Research on the Structure of Matter, Chinese Academy of Sciences, Fuzhou, Fujian 350002, China.

2. School of Chemical Sciences, University of Chinese Academy of Sciences, Beijing 100049, China.

3. Jiangsu Key Laboratory of Biofunctional Materials, School of Chemistry and Materials Science, Ministry-of-Education Key Laboratory of Numerical Simulation of Large-Scale Complex Systems, Nanjing Normal University, Nanjing, Jiangsu 210023, China.

4. College of Chemistry & Chemical Engineering, Weifang University, Weifang, Shandong 261061, China.

5. Physical and Theoretical Chemistry Laboratory, Department of Chemistry, University of Oxford, Oxford OX1 3QZ, United Kingdom.

*Corresponding authors: yanxueyuan@wfu.edu.cn (X. Yan), jiawei.xu@chem.ox.ac.uk (J. Xu) and weihaiyan@njnu.edu.cn (H. Wei)

1. Methodology

All the calculations in this work were performed using the spin-polarized density functional theory (DFT) method of the Vienna Ab initio Simulation Package (VASP).[1] The Perdew-Burke-Ernzerh (PBE) functional of generalized gradient approximation (GGA) was utilized to describe the electron exchange-correlation interaction.[2] The projector-augmented wave (PAW) method was applied to describe the ion-electron interaction and a plane-wave cutoff energy of 450 eV was employed in all calculations. Grimme's DFT-D3 dispersion correction was adopted to consider van der Waals interactions.[3] The Brillouin zone integration was performed with a 1×1×5 grid centered at the gamma (Γ) point.[4] The convergence threshold for energy and force was 10^{-5} eV and 0.02 eV·Å^{-1}, respectively. The space in a- and b-axes was set to 30.0 Å to prevent the interaction introduced by the periodic boundary condition. Crystal orbital Hamilton population (COHP) was used to measure the bonding between atoms by using the concept of the LOBSTER code.[5]

Gibbs free energy of the intermediates was calculated using the computational hydrogen electrode (CHE) model proposed by Nørskov et al.[6] Based on it, the reaction Gibbs free energy (ΔG) of each elementary step can be defined by equation (1):

$$\Delta G = \Delta E - T\Delta S + \Delta \mathrm{ZPE} + \Delta G_U + \Delta G_{\mathrm{pH}} \quad (1)$$

here, ΔE is the electronic energy difference for reactions directly obtained from DFT calculations. ΔZPE and ΔS are the differences between the adsorbed species and the gas phase molecules in zero-point energy and entropy, respectively. $\Delta G_U = -eU$ is the free energy contribution related to applied potential U, which is determined by $U = -\Delta G_{\mathrm{PDS}}/e$, where ΔG_{PDS} is free energy change in potential-determining step (PDS). ΔG_{pH} is refer to the correction of free energy caused by pH ($\Delta G_{\mathrm{pH}} = 2.303k_{\mathrm{B}}\cdot\mathrm{pH}$), and the pH value is set to zero in this work.[7]

The d band center (ε_{d}) is a well-defined electronic descriptor for metal-contained catalysts and is given by equation (2)[8]:

$$\varepsilon_{\mathrm{d}} = \frac{\int n_{\mathrm{d}}(\varepsilon)\varepsilon\, \mathrm{d}\varepsilon}{\int n_{\mathrm{d}}(\varepsilon)\, \mathrm{d}\,\varepsilon} \quad (2)$$

where ε, dε, $n_{\mathrm{d}}(\varepsilon)$ represent energy level, differential of energy, the density of states (DOS) corresponding to energy levels, respectively.

2. Computational results

Table S1. Optimized cell parameters of FeN_4-CNT(m,m) (m = 5 ~ 10). The a and b-axes were fixed to 30.0 Å as vacuum layers.

Catalysts	a (Å)	b (Å)	c (Å)
FeN_4-CNT(5,5)	30.0000000000000000	30.0000000000000000	12.3502075385624916
FeN_4-CNT(6,6)	30.0000000000000000	30.0000000000000000	12.3504509591925267
FeN_4-CNT(7,7)	30.0000000000000000	30.0000000000000000	12.3440500251537184
FeN_4-CNT(8,8)	30.0000000000000000	30.0000000000000000	12.3401455397320543
FeN_4-CNT(9,9)	30.0000000000000000	30.0000000000000000	12.3368985058167677
FeN_4-CNT(10,10)	30.0000000000000000	30.0000000000000000	12.3347005065376880

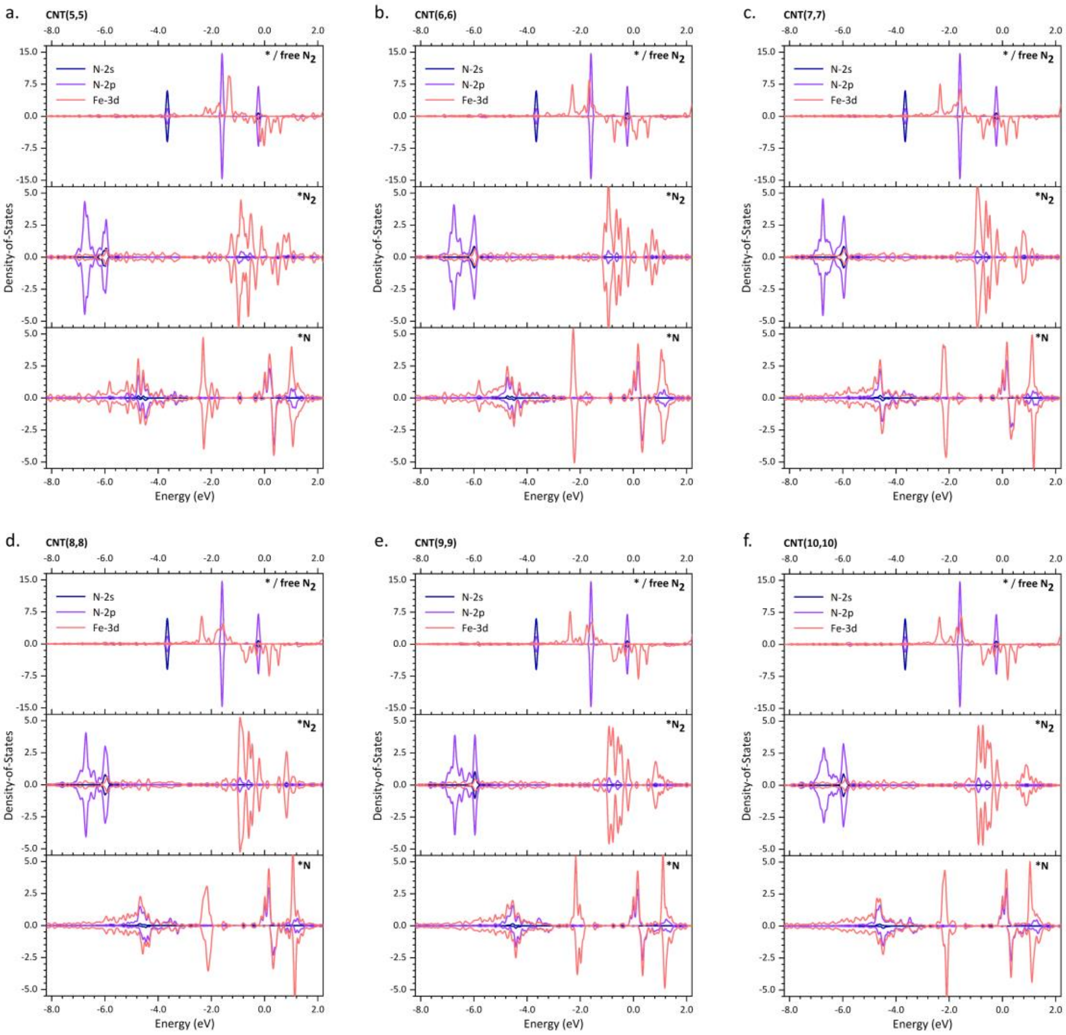

Figure S1. Projected density-of-states (PDOS) results of free N_2, FeN_4-CNT(*m*,*m*), N_2@ and N@FeN_4-CNT(*m*,*m*) (*m* = 5 ~ 10).

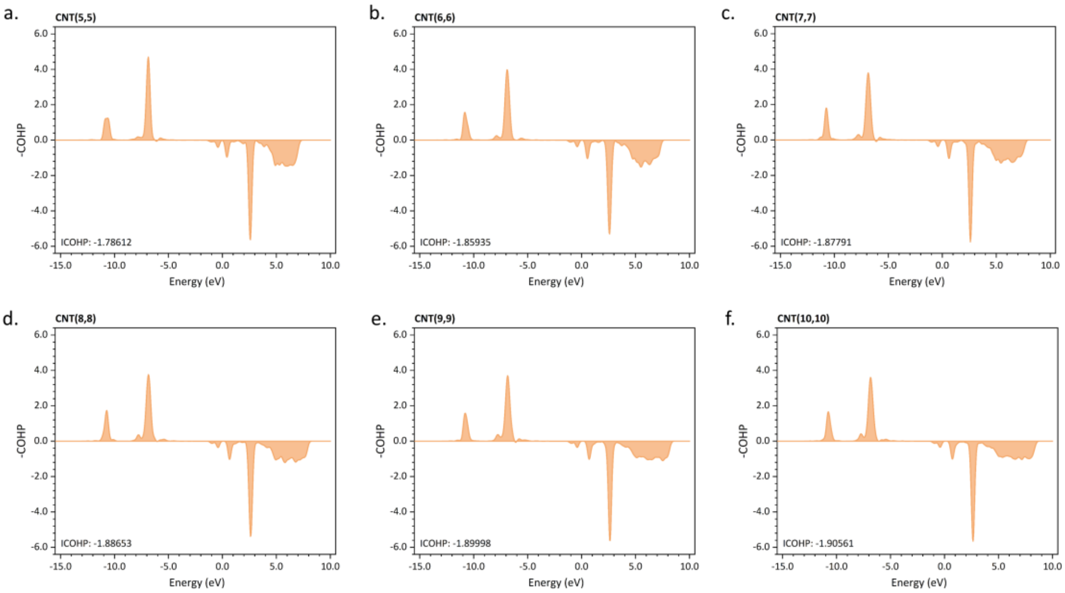

Figure S2. COHP results of N_2@FeN_4-CNT(*m*,*m*) (*m* = 5 ~ 10).

Table S2. Thermochemical data of catalytic intermediates along the alternating pathway of FeN_4-CNT(5,5).

**Energy terms**	**unit**	*	*N_2	*NNH	*NHNH	*$NHNH_2$	*NH_2NH_2	*NH_2NH_3	*NH_2	*NH_3
E_{elc} [a]	a.u.	-890.76360710	-908.21129468	-910.64247763	-914.46821629	-918.01422615	-921.96529498	-926.43685277	-906.71463984	-911.31255875
ZPVE [b]	a.u.	0.000000	0.209522	0.466209	0.826566	1.118724	1.486891	1.608407	0.642762	0.992472
U_{corr} [c]	a.u.	0.000000	0.281323	0.550881	0.911318	1.223769	1.577526	1.789695	0.694007	1.066000
H_{corr} [d]	a.u.	0.000000	0.281323	0.550881	0.911318	1.223769	1.577526	1.789695	0.694007	1.066000
G_{corr} [e]	a.u.	0.000000	0.131774	0.375639	0.733036	1.017874	1.391440	1.388675	0.597931	0.904900
$U(T)$ [f]	a.u.	-890.763607	-907.929972	-910.091597	-913.556898	-916.790457	-920.387769	-924.647158	-906.020633	-910.246559
$H(T)$ [g]	a.u.	-890.763607	-907.929972	-910.091597	-913.556898	-916.790457	-920.387769	-924.647158	-906.020633	-910.246559
$G(T)$ [h]	a.u.	-890.763607	-908.079521	-910.266839	-913.735180	-916.996352	-920.573855	-925.048178	-906.116709	-910.407659

[a] Electronic energy; [b] zero-point vibration energy; [c] thermal correction to energy at 298.15 K; [d] thermal correction to enthalpy at 298.15 K; [e] thermal correction to Gibbs free energy at 298.15 K; [f] thermal energy at 298.15 K; [g] thermal enthalpy at 298.15 K; [h] thermal Gibbs free energy at 298.15 K.

Table S3. Thermochemical data of catalytic intermediates along the distal pathway of FeN_4-CNT(5,5).

**Energy terms**	**unit**	*	*N_2	*NNH	*NNH_2	*NNH_3	*N	*NH	*NH_2	*NH_3
E_{elc} [a]	a.u.	-890.76360710	-908.21129468	-910.64247763	-913.74438069	-917.38087760	-897.81617117	-901.84933228	-906.71463984	-911.31255875
ZPVE [b]	a.u.	0.000000	0.209522	0.466209	0.747290	1.005515	0.083843	0.322391	0.642762	0.992472
U_{corr} [c]	a.u.	0.000000	0.281323	0.550881	0.818050	1.127553	0.114280	0.375494	0.694007	1.066000
H_{corr} [d]	a.u.	0.000000	0.281323	0.550881	0.818050	1.127553	0.114280	0.375494	0.694007	1.066000
G_{corr} [e]	a.u.	0.000000	0.131774	0.375639	0.668039	0.841077	0.061224	0.279653	0.597931	0.904900
$U(T)$ [f]	a.u.	-890.763607	-907.929972	-910.091597	-912.926331	-916.253325	-897.701891	-901.473838	-906.020633	-910.246559
$H(T)$ [g]	a.u.	-890.763607	-907.929972	-910.091597	-912.926331	-916.253325	-897.701891	-901.473838	-906.020633	-910.246559
$G(T)$ [h]	a.u.	-890.763607	-908.079521	-910.266839	-913.076342	-916.539801	-897.754947	-901.569679	-906.116709	-910.407659

[a] Electronic energy; [b] zero-point vibration energy; [c] thermal correction to energy at 298.15 K; [d] thermal correction to enthalpy at 298.15 K; [e] thermal correction to Gibbs free energy at 298.15 K; [f] thermal energy at 298.15 K; [g] thermal enthalpy at 298.15 K; [h] thermal Gibbs free energy at 298.15 K.

Table S4. Thermochemical data of catalytic intermediates along the alternating pathway of FeN_4-CNT(6,6).

Energy terms	unit	*	$*N_2$	*NNH	*NHNH	$*NHNH_2$	$*NH_2NH_2$	$*NH_2NH_3$	$*NH_2$	$*NH_3$
E_{elc} [a]	a.u.	-1078.40833702	-1095.60924674	-1098.02427725	-1101.66905979	-1105.50333029	-1109.34528885	-1113.86262777	-1094.04767317	-1098.48629547
ZPVE [b]	a.u.	0.000000	0.217642	0.467674	0.818018	1.130018	1.497596	1.614642	0.619299	1.012865
U_{corr} [c]	a.u.	0.000000	0.285525	0.550885	0.900108	1.228853	1.582203	1.765757	0.662617	1.098020
H_{corr} [d]	a.u.	0.000000	0.285525	0.550885	0.900108	1.228853	1.582203	1.765757	0.662617	1.098020
G_{corr} [e]	a.u.	0.000000	0.147479	0.377900	0.734371	1.036182	1.416373	1.447469	0.582925	0.905063
$U(T)$ [f]	a.u.	-1078.408337	-1095.323722	-1097.473392	-1100.768952	-1104.274477	-1107.763086	-1112.096871	-1093.385056	-1097.388275
$H(T)$ [g]	a.u.	-1078.408337	-1095.323722	-1097.473392	-1100.768952	-1104.274477	-1107.763086	-1112.096871	-1093.385056	-1097.388275
$G(T)$ [h]	a.u.	-1078.408337	-1095.461768	-1097.646377	-1100.934689	-1104.467148	-1107.928916	-1112.415159	-1093.464748	-1097.581232

[a] Electronic energy; [b] zero-point vibration energy; [c] thermal correction to energy at 298.15 K; [d] thermal correction to enthalpy at 298.15 K; [e] thermal correction to Gibbs free energy at 298.15 K; [f] thermal energy at 298.15 K; [g] thermal enthalpy at 298.15 K; [h] thermal Gibbs free energy at 298.15 K.

Table S5. Thermochemical data of catalytic intermediates along the distal pathway of FeN_4-CNT(6,6).

Energy terms	unit	*	$*N_2$	*NNH	$*NNH_2$	$*NNH_3$	*N	*NH	$*NH_2$	$*NH_3$
E_{elc} [a]	a.u.	-1078.40833702	-1095.60924674	-1098.02427725	-1101.72166331	-1104.72104539	-1085.16901645	-1089.21586399	-1094.04767317	-1098.48629547
ZPVE [b]	a.u.	0.000000	0.217642	0.467674	0.810016	0.995244	0.083930	0.325863	0.619299	1.012865
U_{corr} [c]	a.u.	0.000000	0.285525	0.550885	0.892851	1.049682	0.114450	0.377063	0.662617	1.098020
H_{corr} [d]	a.u.	0.000000	0.285525	0.550885	0.892851	1.049682	0.114450	0.377063	0.662617	1.098020
G_{corr} [e]	a.u.	0.000000	0.147479	0.377900	0.740807	0.932552	0.061130	0.286616	0.582925	0.905063
$U(T)$ [f]	a.u.	-1078.408337	-1095.323722	-1097.473392	-1100.828812	-1103.671363	-1085.054566	-1088.838801	-1093.385056	-1097.388275
$H(T)$ [g]	a.u.	-1078.408337	-1095.323722	-1097.473392	-1100.828812	-1103.671363	-1085.054566	-1088.838801	-1093.385056	-1097.388275
$G(T)$ [h]	a.u.	-1078.408337	-1095.461768	-1097.646377	-1100.980856	-1103.788493	-1085.107886	-1088.929248	-1093.464748	-1097.581232

[a] Electronic energy; [b] zero-point vibration energy; [c] thermal correction to energy at 298.15 K; [d] thermal correction to enthalpy at 298.15 K; [e] thermal correction to Gibbs free energy at 298.15 K; [f] thermal energy at 298.15 K; [g] thermal enthalpy at 298.15 K; [h] thermal Gibbs free energy at 298.15 K.

Table S6. Thermochemical data of catalytic intermediates along the alternating pathway of FeN_4-CNT(7,7).

Energy terms	unit	*	*N_2	*NNH	*NHNH	*$NHNH_2$	*NH_2NH_2	*NH_2NH_3	*NH_2	*NH_3
E_{elc} [a]	a.u.	-1265.00190219	-1282.21321842	-1284.59763244	-1288.29713165	-1291.68562662	-1295.63245898	-1300.46829180	-1280.61111060	-1285.06750241
ZPVE [b]	a.u.	0.000000	0.218216	0.468153	0.828673	1.083541	1.446547	1.617539	0.618000	1.010559
U_{corr} [c]	a.u.	0.000000	0.286233	0.551259	0.892729	1.165828	1.533992	1.792616	0.662164	1.074684
H_{corr} [d]	a.u.	0.000000	0.286233	0.551259	0.892729	1.165828	1.533992	1.792616	0.662164	1.074684
G_{corr} [e]	a.u.	0.000000	0.144973	0.378949	0.763342	0.996807	1.354634	1.416551	0.579737	0.943367
$U(T)$ [f]	a.u.	-1265.001902	-1281.926985	-1284.046373	-1287.404403	-1290.519799	-1294.098467	-1298.675676	-1279.948947	-1283.992818
$H(T)$ [g]	a.u.	-1265.001902	-1281.926985	-1284.046373	-1287.404403	-1290.519799	-1294.098467	-1298.675676	-1279.948947	-1283.992818
$G(T)$ [h]	a.u.	-1265.001902	-1282.068245	-1284.218683	-1287.533790	-1290.688820	-1294.277825	-1299.051741	-1280.031374	-1284.124135

[a] Electronic energy; [b] zero-point vibration energy; [c] thermal correction to energy at 298.15 K; [d] thermal correction to enthalpy at 298.15 K; [e] thermal correction to Gibbs free energy at 298.15 K; [f] thermal energy at 298.15 K; [g] thermal enthalpy at 298.15 K; [h] thermal Gibbs free energy at 298.15 K.

Table S7. Thermochemical data of catalytic intermediates along the distal pathway of FeN_4-CNT(7,7).

Energy terms	unit	*	*N_2	*NNH	*NNH_2	*NNH_3	*N	*NH	*NH_2	*NH_3
E_{elc} [a]	a.u.	-1265.00190219	-1282.21321842	-1284.59763244	-1288.30545824	-1291.30146186	-1271.71733680	-1275.78858420	-1280.61111060	-1285.06750241
ZPVE [b]	a.u.	0.000000	0.218216	0.468153	0.807172	1.006721	0.082684	0.326831	0.618000	1.010559
U_{corr} [c]	a.u.	0.000000	0.286233	0.551259	0.891974	1.126966	0.113532	0.377555	0.662164	1.074684
H_{corr} [d]	a.u.	0.000000	0.286233	0.551259	0.891974	1.126966	0.113532	0.377555	0.662164	1.074684
G_{corr} [e]	a.u.	0.000000	0.144973	0.378949	0.727246	0.851329	0.059535	0.288879	0.579737	0.943367
$U(T)$ [f]	a.u.	-1265.001902	-1281.926985	-1284.046373	-1287.413484	-1290.174496	-1271.603805	-1275.411029	-1279.948947	-1283.992818
$H(T)$ [g]	a.u.	-1265.001902	-1281.926985	-1284.046373	-1287.413484	-1290.174496	-1271.603805	-1275.411029	-1279.948947	-1283.992818
$G(T)$ [h]	a.u.	-1265.001902	-1282.068245	-1284.218683	-1287.578212	-1290.450133	-1271.657802	-1275.499705	-1280.031374	-1284.124135

[a] Electronic energy; [b] zero-point vibration energy; [c] thermal correction to energy at 298.15 K; [d] thermal correction to enthalpy at 298.15 K; [e] thermal correction to Gibbs free energy at 298.15 K; [f] thermal energy at 298.15 K; [g] thermal enthalpy at 298.15 K; [h] thermal Gibbs free energy at 298.15 K.

Table S8. Thermochemical data of catalytic intermediates along the alternating pathway of FeN_4-CNT(8,8).

Energy terms	unit	*	*N_2	*NNH	*NHNH	*$NHNH_2$	*NH_2NH_2	*NH_2NH_3	*NH_2	*NH_3
E_{elc} [a]	a.u.	-1451.10330768	-1468.31605784	-1470.67761986	-1474.58402718	-1477.97137787	-1481.96113719	-1486.55598293	-1466.68871613	-1471.15904730
ZPVE [b]	a.u.	0.000000	0.218941	0.469598	0.835984	1.119377	1.485536	1.618764	0.619629	1.023468
U_{corr} [c]	a.u.	0.000000	0.286824	0.551972	0.915205	1.199342	1.573292	1.793004	0.662589	1.079843
H_{corr} [d]	a.u.	0.000000	0.286824	0.551972	0.915205	1.199342	1.573292	1.793004	0.662589	1.079843
G_{corr} [e]	a.u.	0.000000	0.143656	0.382111	0.759576	1.050642	1.400261	1.416510	0.584334	0.972712
$U(T)$ [f]	a.u.	-1451.103308	-1468.029234	-1470.125648	-1473.668822	-1476.772036	-1480.387845	-1484.762979	-1466.026127	-1470.079204
$H(T)$ [g]	a.u.	-1451.103308	-1468.029234	-1470.125648	-1473.668822	-1476.772036	-1480.387845	-1484.762979	-1466.026127	-1470.079204
$G(T)$ [h]	a.u.	-1451.103308	-1468.172402	-1470.295509	-1473.824451	-1476.920736	-1480.560876	-1485.139473	-1466.104382	-1470.186335

[a] Electronic energy; [b] zero-point vibration energy; [c] thermal correction to energy at 298.15 K; [d] thermal correction to enthalpy at 298.15 K; [e] thermal correction to Gibbs free energy at 298.15 K; [f] thermal energy at 298.15 K; [g] thermal enthalpy at 298.15 K; [h] thermal Gibbs free energy at 298.15 K.

Table S9. Thermochemical data of catalytic intermediates along the distal pathway of FeN_4-CNT(8,8).

Energy terms	unit	*	*N_2	*NNH	*NNH_2	*NNH_3	*N	*NH	*NH_2	*NH_3
E_{elc} [a]	a.u.	-1451.10330768	-1468.31605784	-1470.67761986	-1474.39510623	-1477.41580219	-1457.76628048	-1461.86989116	-1466.68871613	-1471.15904730
ZPVE [b]	a.u.	0.000000	0.218941	0.469598	0.810069	1.012573	0.083519	0.326664	0.619629	1.023468
U_{corr} [c]	a.u.	0.000000	0.286824	0.551972	0.893019	1.152028	0.114184	0.377344	0.662589	1.079843
H_{corr} [d]	a.u.	0.000000	0.286824	0.551972	0.893019	1.152028	0.114184	0.377344	0.662589	1.079843
G_{corr} [e]	a.u.	0.000000	0.143656	0.382111	0.739872	0.829081	0.060566	0.288813	0.584334	0.972712
$U(T)$ [f]	a.u.	-1451.103308	-1468.029234	-1470.125648	-1473.502087	-1476.263774	-1457.652096	-1461.492547	-1466.026127	-1470.079204
$H(T)$ [g]	a.u.	-1451.103308	-1468.029234	-1470.125648	-1473.502087	-1476.263774	-1457.652096	-1461.492547	-1466.026127	-1470.079204
$G(T)$ [h]	a.u.	-1451.103308	-1468.172402	-1470.295509	-1473.655234	-1476.586721	-1457.705714	-1461.581078	-1466.104382	-1470.186335

[a] Electronic energy; [b] zero-point vibration energy; [c] thermal correction to energy at 298.15 K; [d] thermal correction to enthalpy at 298.15 K; [e] thermal correction to Gibbs free energy at 298.15 K; [f] thermal energy at 298.15 K; [g] thermal enthalpy at 298.15 K; [h] thermal Gibbs free energy at 298.15 K.

Table S10. Thermochemical data of catalytic intermediates along the alternating pathway of FeN_4-CNT(9,9).

**Energy terms**	**unit**	*	*N_2	*NNH	*NHNH	*$NHNH_2$	*NH_2NH_2	*NH_2NH_3	*NH_2	*NH_3
E_{elc} [a]	a.u.	-1636.86209767	-1654.06614315	-1656.41737734	-1660.33641438	-1663.72075278	-1667.71188143	-1672.30224468	-1652.42591838	-1656.90633843
ZPVE [b]	a.u.	0.000000	0.220488	0.470981	0.837150	1.122548	1.485594	1.608396	0.618814	1.026182
U_{corr} [c]	a.u.	0.000000	0.287363	0.552846	0.915912	1.225638	1.573203	1.740210	0.662274	1.081423
H_{corr} [d]	a.u.	0.000000	0.287363	0.552846	0.915912	1.225638	1.573203	1.740210	0.662274	1.081423
G_{corr} [e]	a.u.	0.000000	0.149271	0.381223	0.761567	1.013595	1.397841	1.474922	0.581992	0.975657
$U(T)$ [f]	a.u.	-1636.862098	-1653.778780	-1655.864531	-1659.420502	-1662.495115	-1666.138678	-1670.562035	-1651.763644	-1655.824915
$H(T)$ [g]	a.u.	-1636.862098	-1653.778780	-1655.864531	-1659.420502	-1662.495115	-1666.138678	-1670.562035	-1651.763644	-1655.824915
$G(T)$ [h]	a.u.	-1636.862098	-1653.916872	-1656.036154	-1659.574847	-1662.707158	-1666.314040	-1670.827323	-1651.843926	-1655.930681

[a] Electronic energy; [b] zero-point vibration energy; [c] thermal correction to energy at 298.15 K; [d] thermal correction to enthalpy at 298.15 K; [e] thermal correction to Gibbs free energy at 298.15 K; [f] thermal energy at 298.15 K; [g] thermal enthalpy at 298.15 K; [h] thermal Gibbs free energy at 298.15 K.

Table S11. Thermochemical data of catalytic intermediates along the distal pathway of FeN_4-CNT(9,9).

**Energy terms**	**unit**	*	*N_2	*NNH	*NNH_2	*NNH_3	*N	*NH	*NH_2	*NH_3
E_{elc} [a]	a.u.	-1636.86209767	-1654.06614315	-1656.41737734	-1660.13848191	-1663.10331668	-1643.51292235	-1647.61019676	-1652.42591838	-1656.90633843
ZPVE [b]	a.u.	0.000000	0.220488	0.470981	0.810055	1.010492	0.083394	0.325795	0.618814	1.026182
U_{corr} [c]	a.u.	0.000000	0.287363	0.552846	0.893254	1.127467	0.113544	0.376975	0.662274	1.081423
H_{corr} [d]	a.u.	0.000000	0.287363	0.552846	0.893254	1.127467	0.113544	0.376975	0.662274	1.081423
G_{corr} [e]	a.u.	0.000000	0.149271	0.381223	0.738109	0.862414	0.061427	0.287187	0.581992	0.975657
$U(T)$ [f]	a.u.	-1636.862098	-1653.778780	-1655.864531	-1659.245228	-1661.975850	-1643.399378	-1647.233222	-1651.763644	-1655.824915
$H(T)$ [g]	a.u.	-1636.862098	-1653.778780	-1655.864531	-1659.245228	-1661.975850	-1643.399378	-1647.233222	-1651.763644	-1655.824915
$G(T)$ [h]	a.u.	-1636.862098	-1653.916872	-1656.036154	-1659.400373	-1662.240903	-1643.451495	-1647.323010	-1651.843926	-1655.930681

[a] Electronic energy; [b] zero-point vibration energy; [c] thermal correction to energy at 298.15 K; [d] thermal correction to enthalpy at 298.15 K; [e] thermal correction to Gibbs free energy at 298.15 K; [f] thermal energy at 298.15 K; [g] thermal enthalpy at 298.15 K; [h] thermal Gibbs free energy at 298.15 K.

Table S12. Thermochemical data of catalytic intermediates along the alternating pathway of FeN_4-CNT(10,10).

**Energy terms**	**unit**	*	$*N_2$	*NNH	*NHNH	$*NHNH_2$	$*NH_2NH_2$	$*NH_2NH_3$	$*NH_2$	$*NH_3$
E_{elc} [a]	a.u.	-1822.38160915	-1839.58005741	-1841.92641741	-1845.83961939	-1849.22289147	-1853.21866770	-1857.82468825	-1837.93014146	-1842.41650739
ZPVE [b]	a.u.	0.000000	0.221190	0.470934	0.836737	1.117155	1.487627	1.612925	0.619926	1.028349
U_{corr} [c]	a.u.	0.000000	0.287514	0.553265	0.916080	1.199866	1.574583	1.767411	0.662976	1.082221
H_{corr} [d]	a.u.	0.000000	0.287514	0.553265	0.916080	1.199866	1.574583	1.767411	0.662976	1.082221
G_{corr} [e]	a.u.	0.000000	0.152568	0.380877	0.757260	1.041936	1.403396	1.443276	0.583978	0.981761
$U(T)$ [f]	a.u.	-1822.381609	-1839.292543	-1841.373152	-1844.923539	-1848.023025	-1851.644085	-1856.057277	-1837.267165	-1841.334286
$H(T)$ [g]	a.u.	-1822.381609	-1839.292543	-1841.373152	-1844.923539	-1848.023025	-1851.644085	-1856.057277	-1837.267165	-1841.334286
$G(T)$ [h]	a.u.	-1822.381609	-1839.427489	-1841.545540	-1845.082359	-1848.180955	-1851.815272	-1856.381412	-1837.346163	-1841.434746

[a] Electronic energy; [b] zero-point vibration energy; [c] thermal correction to energy at 298.15 K; [d] thermal correction to enthalpy at 298.15 K; [e] thermal correction to Gibbs free energy at 298.15 K; [f] thermal energy at 298.15 K; [g] thermal enthalpy at 298.15 K; [h] thermal Gibbs free energy at 298.15 K.

Table S13. Thermochemical data of catalytic intermediates along the distal pathway of FeN_4-CNT(10,10).

**Energy terms**	**unit**	*	$*N_2$	*NNH	$*NNH_2$	$*NNH_3$	*N	*NH	$*NH_2$	$*NH_3$
E_{elc} [a]	a.u.	-1822.38160915	-1839.58005741	-1841.92641741	-1844.93638112	-1848.60296562	-1828.98678000	-1833.11525679	-1837.93014146	-1842.41650739
ZPVE [b]	a.u.	0.000000	0.221190	0.470934	0.751740	1.012371	0.083371	0.327196	0.619926	1.028349
U_{corr} [c]	a.u.	0.000000	0.287514	0.553265	0.820198	1.128212	0.114113	0.377937	0.662976	1.082221
H_{corr} [d]	a.u.	0.000000	0.287514	0.553265	0.820198	1.128212	0.114113	0.377937	0.662976	1.082221
G_{corr} [e]	a.u.	0.000000	0.152568	0.380877	0.677542	0.870343	0.060321	0.289160	0.583978	0.981761
$U(T)$ [f]	a.u.	-1822.381609	-1839.292543	-1841.373152	-1844.116183	-1847.474754	-1828.872667	-1832.737320	-1837.267165	-1841.334286
$H(T)$ [g]	a.u.	-1822.381609	-1839.292543	-1841.373152	-1844.116183	-1847.474754	-1828.872667	-1832.737320	-1837.267165	-1841.334286
$G(T)$ [h]	a.u.	-1822.381609	-1839.427489	-1841.545540	-1844.258839	-1847.732623	-1828.926459	-1832.826097	-1837.346163	-1841.434746

[a] Electronic energy; [b] zero-point vibration energy; [c] thermal correction to energy at 298.15 K; [d] thermal correction to enthalpy at 298.15 K; [e] thermal correction to Gibbs free energy at 298.15 K; [f] thermal energy at 298.15 K; [g] thermal enthalpy at 298.15 K; [h] thermal Gibbs free energy at 298.15 K.

3. References

(1) Kresse, G.; Hafner, J. Ab initio molecular dynamics for open-shell transition metals. *Physical Review B* **1993**, *48* (17), 13115-13118.

(2) Grimme, S. Semiempirical GGA-type density functional constructed with a long-range dispersion correction. *Journal of computational chemistry* **2006**, *27* (15), 1787-1799.

(3) Blochl, P. E. Projector augmented-wave method. *Physical review B* **1994**, *50* (24), 17953-17979.

(4) Froyen, S. Brillouin-zone integration by Fourier quadrature: Special points for superlattice and supercell calculations. *Physical Review B* **1989**, *39* (5), 3168-3172.

(5) Maintz, S.; Deringer, V. L.; Tchougréeff, A. L.; Dronskowski, R. LOBSTER: A tool to extract chemical bonding from plane-wave based DFT. *Journal of Computational Chemistry* **2016**, *37* (11), 1030-1035.

(6) Nørskov, J. K.; Rossmeisl, J.; Logadottir, A.; Lindqvist, L.; Kitchin, J. R.; Bligaard, T.; Jonsson, H. Origin of the overpotential for oxygen reduction at a fuel-cell cathode. *The Journal of Physical Chemistry B* **2004**, *108* (46), 17886-17892.

(7) Montoya, J. H.; Tsai, C.; Vojvodic, A.; Nørskov, J. K. The challenge of electrochemical ammonia synthesis: a new perspective on the role of nitrogen scaling relations. *ChemSusChem* **2015**, *8* (13), 2180-2186.

(8) Takigawa, I.; Shimizu, K.-i.; Tsuda, K.; Takakusagi, S. Machine-learning prediction of the d-band center for metals and bimetals. *RSC advances* **2016**, *6* (58), 52587-52595. Guo, H.; Li, L.; Wang, X.; Yao, G.; Yu, H.; Tian, Z.; Li, B.; Chen, L. Theoretical investigation on the single transition-metal atom-decorated defective MoS_2 for electrocatalytic ammonia synthesis. *ACS applied materials & interfaces* **2019**, *11* (40), 36506-36514.
